# The Bactericidal Tandem Drug, AB569: How to Eradicate Antibiotic-Resistant Biofilm *Pseudomonas aeruginosa* in Multiple Disease Settings Including Cystic Fibrosis, Burns/Wounds and Urinary Tract Infections

**DOI:** 10.3389/fmicb.2021.639362

**Published:** 2021-06-17

**Authors:** Daniel J. Hassett, Rhett A. Kovall, Michael J. Schurr, Nalinikanth Kotagiri, Harshita Kumari, Latha Satish

**Affiliations:** ^1^Department of Molecular Genetics, Biochemistry and Microbiology, Cincinnati, OH, United States; ^2^Department of Immunology and Microbiology, University of Colorado Health Sciences, Denver, CO, United States; ^3^Division of Pharmacy, University of Colorado Health Sciences, Denver, CO, United States; ^4^Department of Pathology and Laboratory Medicine, University of Cincinnati College of Medicine, Cincinnati, OH, United States; ^5^Shriners Hospitals for Children—Cincinnati, Cincinnati, OH, United States

**Keywords:** bactericidal, AB569, cystic fibrosis, burns/wounds, urinary tract infections

## Abstract

The life-threatening pandemic concerning multi-drug resistant (MDR) bacteria is an evolving problem involving increased hospitalizations, billions of dollars in medical costs and a remarkably high number of deaths. Bacterial pathogens have demonstrated the capacity for spontaneous or acquired antibiotic resistance and there is virtually no pool of organisms that have not evolved such potentially clinically catastrophic properties. Although many diseases are linked to such organisms, three include cystic fibrosis (CF), burn/blast wounds and urinary tract infections (UTIs), respectively. Thus, there is a critical need to develop novel, effective antimicrobials for the prevention and treatment of such problematic infections. One of the most formidable, naturally MDR bacterial pathogens is *Pseudomonas aeruginosa* (*PA*) that is particularly susceptible to nitric oxide (NO), a component of our innate immune response. This susceptibility sets the translational stage for the use of NO-based therapeutics during the aforementioned human infections. First, we discuss how such NO therapeutics may be able to target problematic infections in each of the aforementioned infectious scenarios. Second, we describe a recent discovery based on years of foundational information, a novel drug known as AB569. AB569 is capable of forming a “time release” of NO from *S*-nitrosothiols (RSNO). AB569, a bactericidal tandem consisting of acidified NaNO_2_ (A-NO_2_^–^) and Na_2_-EDTA, is capable of killing all pathogens that are associated with the aforementioned disorders. Third, we described each disease state in brief, the known or predicted effects of AB569 on the viability of *PA*, its potential toxicity and highly remote possibility for resistance to develop. Finally, we conclude that AB569 can be a viable alternative or addition to conventional antibiotic regimens to treat such highly problematic MDR bacterial infections for civilian and military populations, as well as the economical burden that such organisms pose.

## Pseudomonas Aeruginosa

*Pseudomonas aeruginosa* (PA) is an opportunistic pathogen of multiple human infections including but are not limited to cystic fibrosis (CF) airway disease, chronic obstructive pulmonary disease (COPD), wounds (lacerations, abrasions, burns, blast, diabetic), bone, catheter, implant/prosthetic devices, inner ear, urinary tract (UTI), heart valve, and many others. PA is also a member of the six notorious ESKAPE pathogens (*E. faecium, S. aureus, K. pneumoniae, A. baumannii, PA*, and *Enterobacter* sp.), organisms representing those that are multi-drug resistant (MDR) and highly problematic to overall global health. In this review, we will focus on three disease states, CF, burns and UTIs, where MDR-PA is a significant problem pathogen, and within the past several decades, highly refractory to conventional antibiotic regimens.

Despite the relative success of early, aggressive (“eradication”) treatments, *P. aeruginosa* (especially MDR-PA) remains the leading and, arguably, the most formidable pathogen in CF. The majority of individuals with CF becomes chronically infected by adulthood—and although chronic suppressive antimicrobial therapy, primarily with inhaled agents such as tobramycin, aztreonam and colistin, has clear benefits in slowing the progression of lung disease, this intensive antimicrobial exposure drives antibiotic resistance. Approximately one-third of *PA* recovered from adults with CF have an MDR phenotype (Cystic Fibrosis Foundation Patient Registry, 2019 Annual Data Report Bethesda, MD), presenting an enormous challenge to effective antimicrobial therapy, particularly in those with advanced disease.

## Cystic Fibrosis (CF)

**FIGURE 1:**
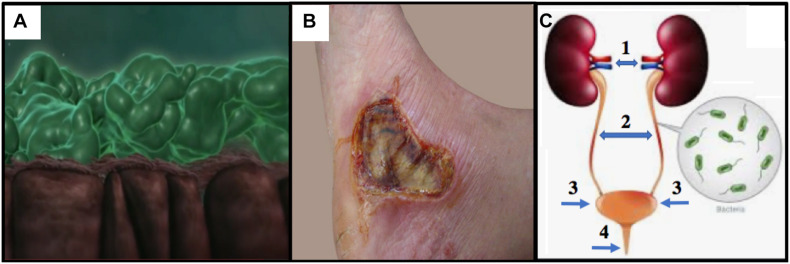
**(A)** CF lung epithelium (brown) with flattened cilia and thick PA Mode II biofilms (green mats) clogging the airway lumen (snapshot from video at https://www.youtube.com/watch?v=YzjnxegMWfk). **(B)** Third degree burn on right foot of male. Note the burn breached the dermis, thereby exposing the hypodermis (from https://www.verywellhealth.com/burn-pictures-4020409). **(C)** The human urinary tract and the potential for (1) pyelonephritis, (2,3) cystitis and (4) urethritis (numbering and arrows added from https://projectheartbeat.com/how-to-a-prevent-urinary-tract-infection-in-the-elderly).

**FIGURE 2:**
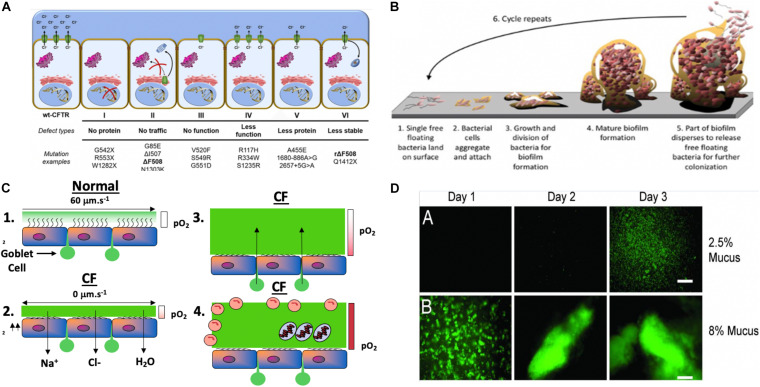
**(A)** Classes of CFTR mutations: a schematic profile (Lopes-Pacheco, 2016). **(B)** Model of Mode I biofilm formation on surfaces, representing the 6 stages of biofilm formation (modified slightly from https://www.google.com/search?source=univ&tbm=isch&q=biofilms&client=safari&sa=X&ved=2ahUKEwihrZjg7eHqAhVKT98KHfyuDPQQiR56BAgHEAw&biw=1505&bih=885#imgrc=GHWUsV_TM0vhXM). **(C)** Model of Mode II *PA* biofilms in hypoxic/anaerobic CF mucus based upon Hassett and Su (Su and Hassett, 2012) relative to normal mucus that was redrawn from Worlitzsch et al. (2002). **(D)** Macrocolony formation and Mode II biofilm formation by GFP-labeled PA in 2.5% **(A)** and 8% **(B)** solids CF mucus (Matsui et al., 2006).

Although a multi-organ disease, CF is most commonly associated with lung abnormalities, particularly CFTR-mediated Cl^–^ secretion across the apical membrane of secretory epithelial cells. CFTR is a multi-transmembrane spanning ABC transporter, the primary function of which is Cl^–^ transport across secretory epithelia (Fenker et al., 2018). Greater than 2000 mutations in *cftr* have been identified (Egan, 2016; Lopes-Pacheco, 2016). These result in several classes of protein dysfunction, affecting CFTR (I) synthesis, (II) maturation, (III) regulation, (IV) conductance, (V) quantity, and (VI) stability (Figure 1; Tildy and Rogers, 2015), The most common mutation is F508del (class II), which exists in ∼70% of CF patients (Rowntree and Harris, 2003). Others such as G542X (class I) and G551D (class III) represent only ∼1–3% of all mutations world-wide (Rogan et al., 2011). Each class of mutations is associated with different degrees of clinical manifestation of CF ranging from severe disease (classes I–III, and VI, (Kerem and Kerem, 1996; Yeh et al., 2019) to moderate or mild disease (classes IV–VI). CFTR class also dictates the quantity and composition of mucins expressed in airways (Thornton et al., 2008) that is likely also mediated by the NPo (number of channels open probability). Generally, CF mucus is 2/3 of the mucins MUC5B and 1/3 MUC5AC in large airways, whereas MUC5B dominates the small airways where the most notable disease is evident. MUC5B is essential for muco-ciliary clearance and bacterial infection control while MUC5AC appears dispensable for the latter functions (Roy et al., 2014). One key feature of normal vs. CF mucus is that the former contains 2% solids, with the latter containing 8–10% (Martens et al., 2011). These mucin proportions represent huge differences of readily available and degradable proteinaceous substrate for metabolism and growth by pathogenic bacteria residing as biofilms within the airway mucus. An underappreciated, yet important feature of this variation in airway mucus is the impact this has on the antibiotic-resistance properties of bacteria residing in the airways in the form of what the corresponding author has coined “Mode II” biofilms (organisms enmeshed in thick mucus as opposed to surface-attached Mode I) (Figure 2B vs. Figure 2A). Metabolism of carbon-rich moieties (including mucin) within such biofilms allows PA (Henke et al., 2011), and many other MDR pathogens such as *Burkholderia cepacia* (Schwab et al., 2014) and *Mycobacterium abscessus* complex (Hunt-Serracin et al., 2019) to thrive in such a niche. The metabolism of mucins in CF can be by serine proteases including those from neutrophils and by PA elastase B (pseudolysin) (Henke et al., 2011) and protease PA3247 (Alrahman and Yoon, 2017). Rather, mucins are fermented by anaerobes in CF to form the readily metabolizable acid, propionate (Flynn et al., 2016), that is readily utilized as a viable carbon source for PA metabolism.

### Problematic Bacterial Infections in CF and a Dearth of Novel Antimicrobials

Despite the tremendous early success of CFTR modifier drug therapy, current evidence indicates that airway infection will continue to be a cause of morbidity and premature mortality in CF. This is especially true for the large proportion of persons with CF whose lung disease preceded the advent of CFTR modifier therapy [for recent review, see Egan (2020)]. In these individuals, particularly those with moderate to severe lung disease, chronic infection, often with MDR-PA bacteria, presents an important and serious challenge to clinical care. Unfortunately, the development of novel antibiotics to meet this challenge has faltered, with no new classes of antibiotics being developed in the last 20 years. Although new combination of β-lactam/ β-lactamase drugs (e.g., ceftazidime/avibactam and meropenem/vaborbactam) showed promise in filling this gap, resistance of bacteria recovered from CF patients to these agents is also on the rise (Caverly et al., 2019).

### Multidrug-Resistant (MDR) Epidemic Strains of *P. aeruginosa* in CF Airway Disease, Hypoxic/Anaerobic Biofilms, and Relationships to Nitric Oxide (NO)

Our seminal past findings (Yoon et al., 2002) coupled with those of Worlitzsch et al. (2002) demonstrated an unappreciated yet important clinical feature of the thick mucus lining the CF airways. We discovered that this mucus is hypoxic or even anaerobic, representing an ideal growth environment for a myriad of opportunistic bacteria that form antibiotic-refractory Mode II biofilms enmeshed in the mucus as “soccerball”-like structures (Figures 2C,D, chronic CF Mode II biofilms; Su and Hassett, 2012). For over 50 years, the most prevalent CF airway pathogen has been *PA*, that exists in about 70–80% of adult patients (O’Toole, 2018), and can grow under both aerobic and anaerobic conditions (Hassett et al., 2004, 2009). Established pulmonary physiology research indicates that nitric oxide (NO), an important antimicrobial component of our innate immune system (Fang, 1997) and potentially toxic bacterial metabolite during anaerobic growth (Yoon et al., 2002), is present at lower levels in CF airways compared to normal individuals (Grasemann et al., 1998; Grasemann and Ratjen, 2012). The diminished production of NO in CF lungs may be an important factor accounting for hypersusceptibility to infections by *PA*. Still, despite such reduced NO levels, its oxidation to the alternative electron acceptors nitrate (NO_3_^–^) and nitrite (NO_2_^–^) likely contributes to microaerobic/anaerobic biofilm growth in the thick mucus (Hassett, 1996; Jones et al., 2000; Palmer et al., 2007a). The importance of these findings, especially in the context of the ever-burgeoning global antibiotic resistance pandemic, is that the most problematic CF infections currently involve multidrug-resistant PA strains (MDR-PA) that often acquire adaptive mutations *in vivo*. Clinically, the presence of such strains often triggers a prolonged course of antibiotics, which have promoted the development of resistance to virtually all antibiotic classes. Thus, the impact of antimicrobial NO, NO donors and AB569 will be discussed later in this review.

## Burn Wounds

Burn wounds represent various breaches of the skin mediated by thermal injury. First degree burns often do not breach the skin, causing some redness, pain, and swelling (e.g., sunburn). In contrast, second degree burns can affect both the epidermis and dermis via contact with stoves, boiling water/oil or fire exposure. Severe pain and infections can ensue, especially when the burn covers large surface areas. Finally, third degree burns essentially remove the dermal and epidermal layers, and perhaps part of the hypodermis. Such burns may surprisingly not be painful, due to extensive nerve damage. These burns may be hard in consistency, be moisture-free, and have a leathery consistency.

Several thousand people die each year because of complications from burn wounds (Mayhall, 2003). Although some burn patients die of burn shock during the first hours, the major course of death is infections, and it is estimated that 75% of all deaths following burns are directly correlated to infection (O’Sullivan and O’Connor, 1997). Burns and wounds occurring in the civilian population are very different from those encountered by the military population. Sir William Osler noted 105 years ago during the artillery war treating casualties that “*shrapnel does the damage, tearing flesh, breaking bones, and always causing jagged, irregular wounds. And here comes in the great tragedy- sepsis everywhere, unavoidable sepsis”* (Osler, 1914). Severe injury of the skin followed by burn/wound infection and sepsis is a serious threat to the life of the patient, as the skin is the largest organ in the body. Sepsis is still a major issue because of its complex pathophysiology (Russell, 2006). Infection leads to complications from sepsis and poses problems to wound healing resulting in significant morbidity. More than 52,000 U.S. military personnel were wounded in action in Iraq [Operation Iraqi Freedom (OIF)] and Afghanistan [Operation Enduring Freedom (OEF)]^1^. The type of infections complicating combat injuries during OIF/OEF was mainly wound infections (84%) followed by blood stream infections (38%). *Acinetobacter* spp. was the most common bacteria associated with infection followed by *Escherichia coli* and *Pseudomonas* spp. (Petersen et al., 2007). However, within a few days, 14–33% of burn wounds are colonized with *PA* (Steinstraesser et al., 2010; Nagoba et al., 2013). *PA* secretes many proinflammatory virulence factors, including elastase, exotoxin A, phospholipase, pyocyanin, rhamnolipid, and homoserine lactones (McCarthy et al., 2014). In addition, this organism possesses a variety of drug resistance mechanisms: inactivation or suppression of enzyme production, increased expression of an active efflux pump system, biofilm formation, and loss or decreased expression of outer membrane proteins or flagella (Livermore, 2002; Lewis, 2008). Burn patients infected with *PA* show a higher mortality rate (Mahar et al., 2010). Biofilms are also an important factor in burn wounds, a process found independent of quorum sensing (QS) (Schaber et al., 2007). It was later found that PA requires.ong chain fatty acids as substrates during acute burn infections (Turner et al., 2014).

Sepsis is often preceded by infectious complications (van Langeveld et al., 2017). Infection is the main cause of delayed wound healing in various types of wounds, including burns (Leaper, 2006). Wound healing is another major issue in the chronology of burn-related disease. Healing is, in part, impeded by the QS-dependent production of PA virulence factors that trigger elevated levels of proinflammatory IL-6, TGF-β, and G-CSF (Rumbaugh et al., 2004). Without rapid and optimal control, patients with severely extensive burns and sepsis can rapidly develop systemic inflammatory response syndrome (SIRS), which results in the damage of many organs, such as the lung, liver and kidney. This leads to the development of multiple organ dysfunction syndrome (Martin et al., 2003; Seymour et al., 2010). Despite improvements in early treatment, survival following burn injury remains challenged, due largely to sepsis, the leading cause of death in both civilian and military populations. As such, antibiotic regimens are predictably and commonly prescribed in the burn population (Rex, 2012). This antibiotic exposure combined with other risk factors increases the risk for acquisition of multidrug-resistant (MDR) bacteria, a global pandemic which poses a formidable threat to human health. These infections are of particular importance to wounded military personnel and veterans, as hospital-associated transmissions of MDR bacteria are common in the settings of surgical sites and trauma units. Thus, given all of the aforementioned issues, burn injuries can frequently result in life-threatening complications (Calhoun et al., 2008). In fact, deaths from drug-resistant infections are projected to increase from 700,000 currently to 10 million annually, and cost estimates are projected to be as high as $100 trillion (US) world-wide by 2050 (Jasovsky et al., 2016). Developing new antibiotics alone cannot fully address the problem, as bacteria inevitably develop resistance to them (Coates and Hu, 2007). Therefore, there have been tremendous efforts to develop alternative approaches to conventional antibiotics for the prevention and treatment of microbial infections. Indeed, the US government has announced a National Strategy to address the problem of antimicrobial resistance^2^. Current topical antimicrobial agents suffer from multiple issues including (a) bacterial resistance (b) lack of penetration into the burn eschar tissues (c) wound healing inhibition and (d) pain upon application that poses adverse systemic effects (Cartotto, 2017). Therefore, it is imperative to develop an agent that can surpass all the aforementioned problems, and can be applied immediately after burn injury in the battlefield and to the civilian patients to prevent biofilm formation while simultaneously promoting wound healing.

## Urinary Tract Infections and *PA*

Urinary tract infections (UTIs) affect an estimated 150 million people worldwide per year and are the leading cause of nosocomial infections in the U.S. In fact, UTIs account for an astounding 40% of all nosocomial infections. Bacterial UTI pathogens include *Escherichia coli*, *Klebsiella pneumoniae*, *Proteus mirabilis*, *Enterococcus* spp., and *PA*. The most frequent cause of problematic UTIs and catheter associated UTI (CAUTI) which is one of the most common infections acquired by patients in health care facilities is *E. coli*, particularly those exhibiting Extended Spectrum β-Lactamase (ESBL) resistance. However, *PA* is associated with chronic UTI especially with CAUTI and most *PA* isolates are resistant to conventional antibiotics used for treatment (Newman et al., 2017). Despite improvements in early therapeutic intervention, increased mortality rates due to antibiotic-resistant bacteria is an ever-burgeoning global health problem. The CDC estimates antibiotic resistant ESKAPE pathogens such as PA cause 2.8 million illnesses and approximately 35,000 deaths per year. *PA* (especially MDR-PA) remains a formidable pathogen in UTIs (Jarvis and Martone, 1992). The CDC considers antibiotic resistance to be among the “biggest threats” because pathogens rapidly evolve new mechanisms to combat drug therapy. *PA* is especially linked with CAUTI and most clinical isolates are resistant to antibiotic treatment regimens. Given these important issues, it is crucial for developing novel antimicrobial agents or alternative tools to combat MDR-PA in UTIs, a huge public health challenge, especially in the highly vulnerable elderly population (Ismail et al., 1997). Bacterial biofilms forming on catheters can cause complicated treatment of CAUTI. Such biofilm catheter infections can lead to pyelonephritis and are nearly impossible to kill because they are up to 1,000-fold more resistant to antibiotics compared to their planktonic (free-living) counterparts due to acquired or inherent antibiotic resistance but also because of poor penetration issues (Stewart, 1996). Generally, the elderly and women are most susceptible to *PA* UTIs and require costly, intensive treatment strategies. The contribution of other host factors and bacterial virulence factors (Newman et al., 2017) to successful infection remains relatively understudied. Thus, there is still a critical and unmet need for effective antimicrobials that (i) prevent, (ii) treat and (iii) eradicate problematic MDR-PA biofilms in UTI’s.

Given the urgent need for the treatment of MDR-PA infections for the aforementioned 3 disease states, we will now shift our course to (i) a brief description of the novel drug, AB569, (ii) to a detailed step-by-step research progression behind the fascinating scientific path that led to its discovery and (iii) some proposed mechanisms of bactericidal action.

## Revisiting No, Its Physiological Sources, and Regulation of Its Production in CF, Burns and Utis

NO itself is antimicrobial, yet it has only a very short half-life in blood (<2 ms). In contrast, NO_2_^–^ has 1.2 million-fold higher half-life (∼40 min, Borlaug et al., 2016) and is reduced to NO from A-NO_2_^–^ (Yoon et al., 2006; Major et al., 2010) or under hypoxic conditions (e.g., heart failure, Simon et al., 2016). The primary sources of NO in the human body are through its generation by three different NO synthases (NOS). Two NOS enzymes, the neuronal NOS (nNOS) and the epithelial NOS (eNOS), are expressed constitutively (Asano et al., 1994). Arguably the most influential with respect to combating pathogen infection is the inducible class of NOS (iNOS) (Fan et al., 1997; Ricciardolo et al., 2004). iNOS is inducible by a number of factors that include bacterial LPS (Nathan and Xie, 1994) and proinflammatory cytokines such as the T-cell generated cytokine IFN-γ (Remy et al., 2017). In CF, iNOS2 activity is dramatically reduced (Wooldridge et al., 2004; Moeller et al., 2006). Such reduced activity is thought to contribute to airway colonization and persistence in the CF airway lumenal mucus. Similarly, patients suffering from burns also have reduced neutrophilic iNOS2 activity (Kartchner et al., 2019). In contrast with CF and burn infections, NO synthase activity was significantly increased during UTI (Smith et al., 1996).

## Phagocyte Contributions of No-Mediated Killing or Growth Inhibition of PA

The respiratory burst of human phagocytic cells NADPH oxidase-catalyzed by the can generate a number of oxygen-centered radicals including the superoxide anion (O_2_^–^), hydrogen peroxide (H_2_O_2_), hydroxyl radical (HO.), singlet oxygen (^1^O_2_) and hypochlorous acid (HOCl). Given that O_2_^–^ is the first radical generated by this process, it can rapidly react with NO in the presence of oxygen to generate highly reactive, diffusion-limited oxidant peroxynitrite (ONOO^–^). In addition to many other cell types in the body, NO can also be generated via iNOS enzymes in both macrophages and neutrophils (MacMicking et al., 1997; Saini and Singh, 2019). However, this event requires accessory contributions by either bacterial LPS or various cytokines (e.g., TNF-α, IL-1, INF-γ). Regarding CF, the versatile chemistry of nitrogen is of central importance to pulmonary physiology, as nitrogen oxidation and reduction occur continuously in both normal and CF lungs. NO is an important antimicrobial component of our innate immune system (Fang, 1997) and a member of what are termed reactive nitrogen species (RNS). Of many conflicting reports, the majority indicate that exhaled NO from CF patients is lower than that produced by normal individuals (Grasemann et al., 1998; Grasemann and Ratjen, 2012). Thus, despite low CF airway NO, one likely reason why concentrations of the dominant CF pathogen, *PA* (Yoon et al., 2002; Palmer et al., 2007b), can approach 10^8^ CFU/ml within microaerobic or anaerobic CF mucus is that it is rich in the alternative electron acceptors NO_3_^–^ (383 ± 42 mM) and NO_2_^–^ (125 ± 55 mM) (Jones et al., 2000), levels which are 2–3 fold higher than in normal individuals (Hassett, 1996; Palmer et al., 2007a).

## How Does PA Sense and Combat No?

Arguably, the most influencing proteins involved in NO metabolic are enzymes which detoxify it, including NOR (Arai et al., 1995) and the hemoglobin/NO dioxygenase, Hmp (Yoon et al., 2007). As expected, bacteria lacking NOR grow abysmally slowly under anaerobic conditions (Yoon et al., 2007). This is due to the NO-mediated inactivation of the 4Fe-4S cluster of the master anaerobic regulator, ANR (Yoon et al., 2007). Without ANR, the NO-responsive second-tier regulator, DNR, is incapable of activating downstream *nar*, *nir*, *nor*, and *nos* genes (Schreiber et al., 2007). Elegant recent work by Boon and colleagues have illuminated previously unrecognized NO binding proteins (Hossain and Boon, 2017; Bacon et al., 2018; Williams et al., 2018; Williams and Boon, 2019). NosP, an NO-sensing protein, was discovered by Boon and colleagues as a critical protein involved in the dispersal of mature PA biofilms (Hossain and Boon, 2017). NosP was actually a protein that was anticipated to exist, for the majority of bacteria NO sensing protein are of the H-NOX variety (Williams et al., 2018; Williams and Boon, 2019). NosP is also a regulator of a histidine kinase signal transduction pathway that paradoxically is also critical for optimal biofilm formation (Williams et al., 2018).

## No Generators and Control And/Or Killing of PA in Biofilms

NO, or their chemical generators, have also been used in the treatment of biofilms from two different fronts. First, there is the antimicrobial end. NO itself or compounds that generate it by hydrolysis or reduction, is capable of killing not only PA biofilms, but also a myriad of other problematic pathogens. Regarding biofilm control, dispersal and/or killing of resident bacteria, many compounds that are not NO or acidified nitrite have been used. For examples, please refer to Table 1. In an *ex vivo* model of PA dispersion, submicromolar NO disrupted biofilms in CF sputum (Howlin et al., 2017). Another example Barraud and colleagues ion 2006 (Barraud et al., 2006) demonstrated that NO-generating compounds are capable of dispersing mature PA biofilms. The addition of NO release significantly improved the antibiofilm action of the hyperbranched polymers, with NO-releasing hyperbranched polyamidoamines of largest NO payloads being more effective than hyperbranched polykanamycins. Furthermore, the NO-releasing hyperbranched polymers reduced the biofilm metabolic activity in a dose-dependent manner, killing biofilm-detached bacteria under both aerobic and anaerobic conditions, with greater antimicrobial efficacy observed under aerobic conditions (Yang et al., 2020). Other similar NO-generating compounds include sodium nitroprusside (SNP, Barraud et al., 2006), S-nitrosoglutathione (GSNO Neufeld and Reynolds, 2016), spermine NON-Oate (Cai and Webb, 2020), NO-releasing alginates (Ahonen et al., 2018), and chitosan oligosaccharides (Reighard et al., 2015). Cephalosporin-based NO donor prodrugs (cephalosporin-3’-diazeniumdiolates) not only release NO, but possess direct β-lactam mediated antibacterial activity and antibiofilm effects (Yepuri et al., 2013; Rineh et al., 2020). Cephalosporin NO-donor prodrug DEA-C3D (diethylamin-cephalosporin-3′-diazeniumdiolate) has also been shown to disperses PA biofilms formed by cystic CF isolates (Soren et al., 2020). Finally, additional biocompatible compounds oligoethylene glycol, hydrophobic ethylhexyl, cationic primary amine, and nitric oxide (NO)-releasing functional groups have been not only shown to disperse mature biofilms, but also puncture membranes (18).

**TABLE 1:** NO, A-NO_2_^–^, GSNO, AB569, and other NO-related anti-pseudomonas compounds and their effects on *P. aeruginosa*.

Agent	Organism	Growth/Biofilm inhibition	Killing	Biofilm dispersion	References
Acidified nitrite	*P. aeruginosa* PAO1 *P. aeruginosa* PAO1 *mucA22 P. aeruginosa* FRD1 *mucA22*	Yes/ND	Yes	ND	Yoon et al., 2006; Major et al., 2010; McDaniel et al., 2016, 2020
Acidified nitrite	*P. aeruginosa* PAO1	Yes/Yes	Yes	ND	Major et al., 2010
NO donor (SNP)	*P. aeruginosa* PAO1	ND	ND	Yes	Barraud et al., 2006
EDTA	*P. aeruginosa* PAO1	Yes/Yes	Yes	Yes	Banin et al., 2006
Na4-EDTA	*P. aeruginosa*		Yes		Percival and Salisbury, 2018
Acidified Nitrite + Na_2_-EDTA	All tested Gram-negative and Gram-positive bacteria	Yes/Yes	Yes	Yes	McDaniel et al., 2020
S-nitrosoglutathione (GSNO)	*P. aeruginosa*	ND/ND	Yes	ND	Neufeld and Reynolds, 2016
Spermine NON-Oate, Sodium nitroprusside	*P. aeruginosa*	Yes/Yes	No	Yes	Cai and Webb, 2020
NO-releasing alginates	*P. aeruginosa*	ND/ND	Yes	ND	Ahonen et al., 2018
DEA-C3D (diethylamin-cephalosporin-3’-diazeniumdiolate)	*P. aeruginosa*	ND	ND	Yes	Yepuri et al., 2013
	*P. aeruginosa*	Yes	ND	Yes	Rineh et al., 2020
NO-releasing chitosan oligosaccharides	*P. aeruginosa* PAK *fliC pilA, mucA22*	Yes/Yes	ND	Yes	Reighard et al., 2015

**ND, not determined.**

## Why Inhaled or Topical No Is Likely Not Nearly as Effective as AB569

First, inhaled NO can rapidly react with O_2_ in the lung to form nitrogen dioxide (NO_2_), a known potent pulmonary irritant. Second, the vast majority of inhaled NO is exhaled. In the case of pulmonary hypertension, inhaled NO was considered a potential therapy, yet the short half-life of NO requires essentially constant inhalation for a sustained positive effect (Amdahl et al., 2019). In infected airways, AB569 would be aerosolized as a ***mist***. In the case of CF, the mucus pH is already acidic (pH 6.3–6.5) where a buffer is not required, thus triggering the production of NO **within** (and not above) the infected airway mucus. In the case of topical use of AB569, the A-NO_2_^–^ component has been used safely to kill other organisms (Weller et al., 1998; Ormerod et al., 1999; Phillips et al., 2004; Jowkar et al., 2010). An A-NO_2_^–^ emollient or cream at 430 mM NaNO_2_ has been shown to improve the rate and extent of incision wound healing in normal and diabetic mice (Weller and Finnen, 2006). We have shown that A-NO_2_^–^ (McDonald and Thornton, 1994; McDaniel et al., 2016, 2020), the NO donor sodium nitroprusside (SNP) (Barraud et al., 2009), and an absence of endogenous NO reductase (NOR) (Barraud et al., 2009), triggers PA biofilm dispersion, rendering such organisms now vastly more susceptible to antibiotics. Similarly, the amount of EDTA typically used in nebulized bronchodilators does not induce bronchospasm (Asmus et al., 2001). Some additional benefits of EDTA is that it can disperse and kill mature PA in biofilms (Banin et al., 2006) and can achieve complete eradication of 7 bacterial pathogens (including 5 ESKAPE members) in skin, bone, implant infections (Deng et al., 2019). In UTI’s, based upon the low pH of morning voided urine, the acidification of nitrite has been shown to kill nitrate reducing bacteria such as *E. coli* and *PA* (Carlsson et al., 2003, 2005). Similarly, Mir and colleagues recently showed that the 3-pronged combination of ceftriaxone, sublactam, and Na_2_-EDTA killed infectious bacteria caused by extended spectrum β-lactamase (ESBL)-producing Gram-negative bacteria (Mir et al., 2019).

## The Experimental Chronology Leading to the Discovery of AB569, an Alternative to Strictly No-Based Therapeutics

### Two Epidemic Mutant Strains of *PA* Emerge During CF Lung Disease: *lasR/rhlR* QS and Mucoid *mucA* Clinical Isolates

*PA*, like many Gram-negative and Gram-positive bacteria, engages in a form of intercellular communication known as quorum sensing (QS) (Whitehead et al., 2001). QS in *PA* regulates many bacterial processes including virulence factor production (Gambello and Iglewski, 1991; Brint and Ohman, 1995) and animal (Rumbaugh et al., 1999), nematode (Papaioannou et al., 2009), and insect (Park et al., 2014) pathogenesis. *PA* encodes two acyl-homoserine lactone (AHL) QS circuits, LasI/LasR and RhlI/RhlR. During *in vivo* evolution in early CF, *lasR/rhlR* mutants in QS circuitry are frequently isolated (Bjarnsholt et al., 2010; Feliziani et al., 2010), especially in the early stages of the disease. The Liverpool epidemic strain, LES400, is a *lasR* mutant that overproduces the redox-active virulence factor pyocyanin, is highly antibiotic-resistant, and is hyper-virulent (Salunkhe et al., 2005). In addition to QS inactivation, CF isolates during late-stage chronic disease are often mucoid (alginate overproduction) during the late, chronic disease stages, resulting from mutations in the anti-sigma factor gene, *mucA* (Martin et al., 1993; Bjarnsholt et al., 2010; Feliziani et al., 2010). Alginate is a highly viscous exopolysaccharide the production of which causes a precipitous decline in overall airway oxygen processing (Sanderson et al., 2008). Below is an intriguing chronology of why we believe that *lasR/rhlR* and ultimately *mucA* mutants emerge during the course of CF lung disease—a direct connection to the low nitric oxide (NO) levels produced in CF lung disease. Finally, the diminished production of innate NO in CF lungs may be an important factor accounting for hypersusceptibility to infection by *lasR/rhlR* and *mucA* mutant *PA* strains *in vivo*.

### *lasR* and *rhlR* QS Mutants Perish in Anaerobic Biofilms Due to Overproduction of Endogenous, Metabolic Nitric Oxide (NO)

**FIGURE 3:**
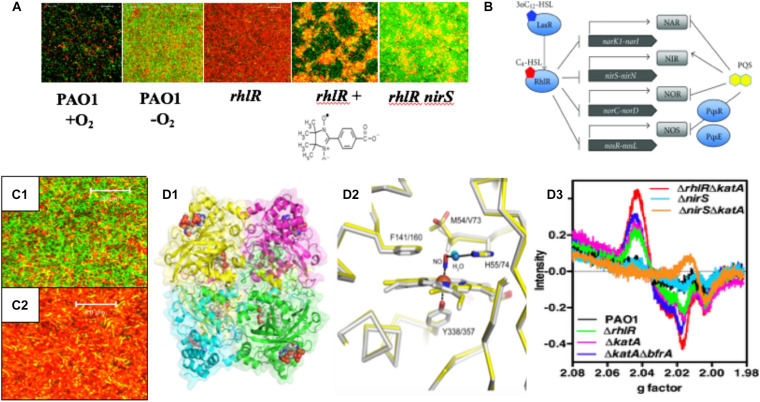
**(A)** PA forms more robust biofilms under anaerobic conditions and *rhlR* mutants die in anaerobic biofilms due to overproduction of endogenous NO. The NO scavenger, C-PTIO (chemical structure below *rhlR* biofilm) and inactivation of *nirS* protects *rhlR* mutant bacteria from NO-mediated death (Yoon et al., 2002). **(B)** PA RhlR is a negative regulator of the anaerobic denitrification genes *nar*, *nir*, *nor* and *nos* (Toyofuku et al., 2012). **(C1)** Close-up version of anaerobic wild-type biofilms noting the long rods, the majority of which are viable. **(C2)** Close-up of dead *rhlR* mutant biofilms. Note that the individual cellular integrity seems to be exploding, with little semblance of the structures of wild-type bacteria. **(D1)** Ribbon diagram and cpk model of hemes of the structure of the major NO-buffering catalase in PA KatA. **(D2)** Methionine sulfone group 5 Å from the NO in KatA (Su et al., 2014). **(D3)** Enhancement of dintrosyliron (DNIC) formation in a *rhlR katA* mutant (red line) relative to *rhlR* (green line) and wild-type bacteria (black line).

A seminal study in 1998 performed under aerobic conditions indicated that QS was essential for optimal biofilm formation, matrix development and maturation (Davies et al., 1998). We coined such surface attached bacterial structures as Mode I biofilms (Su and Hassett, 2012). A *lasI/rhlI* mutant was found to produce thin and densely packed biofilms relative to the mature, well-developed biofilms of wild-type bacteria. In contrast, those enmeshed within the thick CF mucus are termed Mode II (Su and Hassett, 2012) biofilms (Figures 2A,B). In chronic CF lung disease, there is progressively poor airway oxygenation, resulting in what is termed pulmonary insufficiency (Elborn, 2016). Such events trigger markedly reduced oxygen tension for both the host and resident airway bacteria such as *PA* (Worlitzsch et al., 2002; Yoon et al., 2002). Until 2002, biofilm formation in the context of chronic CF airway disease was poorly understood, especially the role of oxygen tension. Subsequently, Worlitzsch et al. (2002) and Yoon et al. (2002) demonstrated not only hypoxic gradients within the thick mucus, but also anaerobic conditions. Given these discoveries coupled with the near crippling CF airway oxygenation of chronic CF patients, we elected to examine the role of QS under strict anaerobic conditions. First, wild-type bacteria were discovered to form far better (>3-fold) biofilms during anaerobic relative to aerobic growth (Figure 3A). When a *rhlR* mutant was grown anaerobically as biofilms virtually all organisms were dead, an event that initially puzzled us until we revisited the anaerobic respiratory pathway of PA. Although nitrate and nitrite can serve as terminal electron acceptors during anaerobic growth and have ATP-generating coupling steps, the remaining 3 gases produced are NO, nitrous oxide (N_2_O) and N_2_. Because NO can be powerfully antimicrobial, our first hypothesis was that the *lasR* and *rhlR* mutants perished due to overproduction of endogenous NO in biofilms. Surprisingly, *rhlR* mutant bacteria generated nearly 40-fold higher endogenous NO than wild-type bacteria resulting in anaerobic biofilm death. This was proven first biochemically when we added C-PTIO, a NO scavenger, that protected the bacteria. This was followed genetically when we created a *rhlR nirS* double mutant that is incapable of producing endogenous NO that was viable in anaerobic biofilms. With this discovery, it became obvious that paralysis of anaerobic QS could be one mechanism to kill PA during chronic CF.

Our previous work demonstrates that *rhl* QS is critical for anaerobic biofilm survival as *rhlR* mutant bacteria die as a result of overproduction of endogenous metabolic NO (Yoon et al., 2002). Still, it was unclear how the inactivation of *rhl* QS results in increased levels of endogenous NO that result in killing. Finally, our results became clearer when it was discovered that RhlR represses the anaerobic denitrification genes *nar*, *nir*, *nor*, and *nos* (Toyofuku et al., 2007, 2012; Figure 3B). Upon revisiting the overall bacterial integrity on anaerobic biofilms, we observed that wild-type bacteria had elongate rods and were mostly viable (Figure 3C1). In contrast, the integrity of bacteria on *rhlR* mutant biofilms almost represented a “war zone,” with what appears to be organisms literally exploding, leaving only a bacterial “skeleton” or “corpse” (Figure 3C2). An additional factor involved in anaerobic biofilm survival is the major catalase, KatA (Ma et al., 1999; Hassett and Imlay, 2006). KatA is a tetrameric catalase (Figure 3D1) that is responsive to both H_2_O_2_ in a QS (Hassett et al., 1999) and OxyR-dependent (Heo et al., 2010) fashion and anaerobic conditions in an ANR-dependent fashion (Trunk et al., 2010; Su et al., 2014; Kim et al., 2019). In each monomer, KatA has a methionine sulfone group that helps “buffer” NO (Figure 3D2), thus protecting it from NO generated from A-NO_2_^–^ (Su et al., 2014). When we constructed a *rhlR katA* double mutant, we observed high levels of damaging dinitrosyliron complexes (DNICs, Su et al., 2014), far more than that of the wild-type and *rhlR* mutant alone (Figure 3D3). Thus, the logical correlate from an absence of RhlR is a dramatic overproduction of lethal NO. When coupled with an absence of KatA, anaerobic *rhlR katA* bacteria are severely stressed by substantially elevated NO during anaerobic biofilm growth, resulting in overproduction of NO and concomitant DNICs, resulting in cell death.

### Mucoid, *mucA* Mutant Bacteria Have an Inherent Sensitivity to Acidified Nitrite, an NO Generator Under the Acidic Conditions of the CF Airway Mucus

**FIGURE 4:**
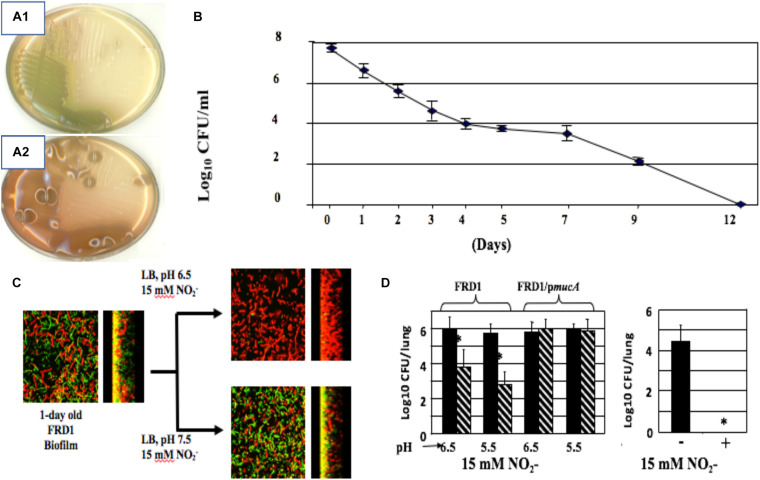
Non-mucoid **(A1)** and mucoid **(A2)** bacteria. **(B)** A-NO2^–^ treatment of mucoid bacteria for a period of 12 days kills all organisms with no resistance developed throughout the course of exposure. **(C)** Mucoid *mucA22* CF isolate FRD1 was grown as biofilms on glass surfaces and exposed to A-NO2^–^ at pH 6.5 and 7.5. Note that all bacteria are dead (red stain, propidium iodide) at pH 6.5, while a mixture of live (Green, Syto9 stain) and dead were observed at pH 7.5. **(D)** Killing of PA FRD1 *mucA22* in a chronic lung infection model in mice and protection by the *mucA* gene *in trans*. Right caption. All *mucA22* bacteria were dead after 16 days of exposure to A-NO2^–^ at pH 6.5. *repesents statistical significance (*P* < 0.01). **(B–D)** are re-worked and re-formatted from Yoon et al. (2006) for this review.

In 2006, we discovered that alginate-overproducing *mucA* mutants (Figure 4A2 vs. nonmucoid 4A1) devoid of the anti-sigma factor MucA are more susceptible to exogenous NO produced by acidified nitrite (A-NO_2_^–^) than wild-type bacteria at the pH of the CF airway mucus (Yoon et al., 2006). This is under planktonic (Figure 4B), biofilm (Figure 4C), and during chronic infection in mice (Figure 4D). However, unlike the mechanism underlying endogenous NO-mediated biofilm death of *rhlR* mutant bacteria described above, *mucA* mutant bacteria are sensitive to exogenous NO mediated by A-NO_2_^–^ reduction (Yoon et al., 2006). Inactivation of *mucA* allows for the sigma factor, AlgT(U) to regulate and transcribe genes involved in alginate production (Martin et al., 1993). However, after scanning the literature for potential effects of AlgT(U) on anaerobic respiratory genes, we only found evidence that a *mucA22 sup-2* mutant that generates reduced alginate production demonstrated increased transcription of *norB*, *norC*, and *norD* (Firoved et al., 2004). The identity of the *sup-2* mutation remains unknown. Furthermore, *mucA22* mutants lacking AlgD (no alginate) or AlgT(U) were still sensitive to A-NO_2_^–^. Still, the reason why *mucA22* mutant bacteria are sensitive to A-NO_2_^–^ was only elucidated after examining anaerobic NAR, NIR and NOR activities in *mucA22* vs. wild-type bacteria. We noted an ∼16-fold reduction in NIR and 4.5-fold reduction in NOR activity (Yoon et al., 2006).

### How Else Might *las/rhl* and *mucA* Mutants Die From NO Derivatives? the Potential Relationship Between *PA* Denitrification, Biofilms, and Filamentous Pf Bacteriophages

Bacteria respond to nitrosative and oxidative stress by activating hundreds of genes. In *PA*, one of these genes is *oxyR*, which encodes a LysR-type transcriptional regulator that positively regulates several dozen genes involved in RNS (e.g., RSNO) and ROS (O_2_^–^, H_2_O_2_, HO., ^1^O_2_) metabolism, such as catalases, alkylhydroperoxide reductases and superoxide dismutases (Ochsner et al., 2000; Vazquez-Torres, 2012; Wei et al., 2012). OxyR also binds to the Pf4 prophage in the intergenic region between *PA0716* and *PA0717* (Wei et al., 2012), suggesting that both nitrosative and oxidative stress may induce Pf4.

**FIGURE 5:**
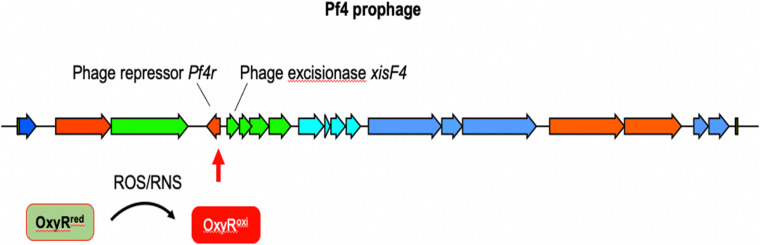
The12.4 kbp Pf4 prophage is integrated into the PAO1 chromosome at the tRNA-Gly gene *PA0729.1*. OxyR binds the motif ATAGAGCAAGACTAT present in the 3′ end of the repressor c gene *Pf4r* (Cobessi et al., 2005b; Wei et al., 2012). It is not clear if OxyR affects *Pf4r* or *xisF4* expression; presumably OxyR positively regulates *xisF4*, which induces prophage excision and initiation of Pf4 virion production.

In contrast to lytic phages that must lyse and kill the host bacterium to complete their lifecycle, filamentous Inoviruses such as Pf4 (Figure 5) are extruded from the cell without causing lysis (Rakonjac et al., 2011; Mai-Prochnow et al., 2015; Secor et al., 2020). However, *PA* biofilms routinely generate superinfective Pf4 variants that are capable of lysing their host. Pf4-induced cell lysis occurs in the center of biofilms where anaerobic conditions necessitate the use of nitrogen oxides (e.g., NO_3_^–^, NO_2_^–^) as alternative electron acceptors, which produces RNS in the form of NO (Hassett et al., 2004; Yoon et al., 2007; Arai, 2012). We have shown that Pf4 genes are some of the most activated genes when PA is grown under anaerobic conditions using NO_3_^–^ or NO_2_^–^ as terminal electron acceptors (Platt et al., 2008). After induction, the Pf4 excisionase XisF4 promotes the transcription of the recombinase IntF, excising the prophage from the chromosome forming a circular dsDNA species called the replicative form (RF) (Li et al., 2019). The RF replicates via rolling circle replication producing one dsDNA and one ssDNA copy (Martinez and Campos-Gomez, 2016). The ssDNA species can either be converted into a dsDNA RF or packaged into filamentous virions. When Pf4 is actively replicating, mutations in the phage repressor C gene *Pf4r* occur at high frequencies (McElroy et al., 2014). These mutations are thought to inactivate the repressor, resulting in a runaway superinfection that lyses *PA*. Cell lysis increases levels of extracellular DNA and the emergence of superinfective Pf4 variants is associated with biofilm dispersal, both important components of the biofilm lifecycle (Webb et al., 2003, 2004; Rice et al., 2009; Petrova et al., 2011).

In the laboratory, *PA* biofilms consistently produce as many as 10^10^ infectious virions per ml (Webb et al., 2003, 2004; Rice et al., 2009; McElroy et al., 2014). As these filamentous virions accumulate in the biofilm matrix, they spontaneously align and assemble a liquid crystal (Secor et al., 2015a). The highly ordered liquid crystalline matrix promotes water retention, allowing *PA* to better withstand desiccation (Secor et al., 2015a). Pf4 virions also bind and sequester cationic antimicrobials away from *PA*, increasing antimicrobial tolerance and ensuring viability of its host (Janmey et al., 2014; Secor et al., 2015a,b). In animal models of infection, Pf4 phages increase the virulence potential of *PA* in both lung (Rice et al., 2009) and wound infection models (Sweere et al., 2019). Recent work demonstrates that Pf4 virions have immunomodulatory properties that steer the infection response toward a maladaptive type I interferon antiviral response that suppresses phagocytic uptake of bacteria, promoting infection initiation (Sweere et al., 2019).

Pf4 and related Pf prophages are prevalent amongst *PA* clinical isolates (Mooij et al., 2007; Knezevic et al., 2015; Burgener et al., 2019; Secor et al., 2020) and Pf virions are produced in abundance at sites of human infection (Secor et al., 2015b; Burgener et al., 2019; Sweere et al., 2019). Pf phages represent a therapeutic target to treat or prevent *PA* infections, especially were AB569 to trigger Pf4 excision events. Indeed, vaccinating against the Pf4 coat protein prevents *PA* infections in mice (Sweere et al., 2019). Future studies exploring the relationship between the anaerobic respiration (e.g., denitrification) processes and Pf phage induction may reveal additional therapeutic strategies to treat *PA* infections.

### A New Clue to the Puzzle That Helped the Discovery of AB569: PA4455, a Putative ABC Transporter Permease

In 2016, we were interested in additional genes involved in A-NO_2_^–^ sensitivity. We embarked on a screen of previously constructed isogenic and Tn mutants of *PA* PAO1. Among several mutants that were sensitive to A-NO_2_^–^, the most prominent including *mucA* (described above) and *PA4455*. The latter strain was devoid of PA4455, encoding a 6-membrane spanning ABC transporter permease (McDaniel et al., 2016) which was also found to be sensitive to the membrane perturbing agent EDTA. In fact, killing of both *PA* PAO1 and especially the *PA4455* mutant was even more sensitive to A-NO_2_^–^ plus EDTA. Since EDTA can destabilize the LPS layer of the outer membrane, specifically by stripping the structurally essential cations Ca^2+^ and Mg^2+^, we also examined sensitivity of various A-band and/or B-band mutants. Two B-band LPS mutants *wbpM* (A^+^, B^–^) and especially *rmlC* (A^–^, B^–^) were susceptible to A-NO_2_^–^, EDTA and especially A-NO_2_^–^ + EDTA under both aerobic and even more so under anaerobic conditions (McDaniel et al., 2016). Interestingly, the *rmlC* mutant could not grow anaerobically (McDaniel et al., 2016).

Upon the discovery of the *PA4455* mutant being uniquely susceptible to both A-NO_2_^–^ and EDTA, we literally raided our collection of nearly 6000 bacterial frozen stocks for both Gram-negative and Gram-positive bacterial pathogens, especially those that were deemed MDR. First, however, we found two PA MDR strains called “Andrea” and “BAMF” (McDaniel et al., 2020). The Andrea strain was isolated from a woman with a ΔF508 that passed away at 35 years of age that was resistant to every antibiotic except colistin. In contrast, the BAMF strain was isolated from an 8-year-old girl after a small bowel-liver transplantation that was resistant to all antibiotics including colistin. Both had fractional inhibitory concentrations values of ∼0.5, indicative of synergy between A-NO_2_^–^ and EDTA. A transcriptomic and rigorous biochemistry and biophysics-based research strategy rapidly ensued using *PA* as a model organism and anaerobic conditions, representative of chronic, late-stage CF airway disease (Worlitzsch et al., 2002; Yoon et al., 2002). First, using RNA-seq analysis, we found a dramatic downregulation of a plethora of essential genes involving the biosynthesis of DNA, RNA, protein and ATP (Figure 5, red minus sign) coupled with a significant upregulation of genes involved in iron acquisition (Figure 5, green plus sign). In addition, we unexpectedly found a decrease in the transcription of the *fur* gene, encoding the ferric uptake regulator, by treatment with anaerobic A-NO_2_^–^ alone. In *E. coli*, purified iron (Fe)-Fur to form a S = 1/2 low-spin Fe-Fur–NO complex with a *g* = 2.03 EPR signal (D’Autreaux et al., 2002). However, NO derived from the HONO generated by A-NO_2_^–^ could react with Fe^2+^ in the Fur protein, thereby creating a flux of Fe^2+^-Fur and Fe^3+^-Fur, resulting is a derepression of genes that are normally tightly repressed by Fe^2+^-Fur such as the *fpvA* and *ftpA* genes encoding the ferripyoverdin and ferripyochelin receptors, respectively (Ochsner and Vasil, 1996; Figure 5, right panel, ribbon structures) as well as a myriad of siderophore biosynthetic and regulatory genes (McDaniel et al., 2020).

Regarding the chemistry of AB569, we have only just scraped the surface. Using three different techniques including NO polarographic measurements, cyclic voltammetry and electron paramagnetic resonance spectroscopy, we found a number of interesting features of AB569. However, these studies were only performed as yet in laboratory media or buffered solutions and not in human-relevant reagents such as airway surface liquid (e.g., CF/COPD), blood or serum (e.g., burns/wounds) or urine (e.g., UTIs). Thus, the mechanistic effect of AB569 at various oxygen tensions and human tissues remains a mystery. Still, we offer some glimpses of planned future work involving AB569 mechanism. Obviously, RNA-seq analysis of maximally down-regulated genes revealed in organisms exposed to AB569 in respective fluids coupled with an isogenic mutant analysis and followed by AB569 sensitivity measurements would be a logical start. Because EDTA can redox-cycling iron from the Fe^2+^/Fe^3+^ couple, substitution of diethylenetriamine pentaacetic acid (DTPA) which only chelates the reduced form for EDTA would indicate that redox-cycling is required for bactericidal activity if DTPA is not synergistic. Second, oxygen tension is likely also a major player in AB569 activity. For example, human urine has ∼55 mM O_2_, where oxygen saturated water is 260 mM (Jalali et al., 2009).

### AB569: A Novel Bactericidal Agent Against All MDR Gram-Negative and Gram-Positive Bacteria Including the ESKAPE Pathogens

Finally, AB569 is an innovative bactericidal combination of acidified nitrite (A-NO_2_^–^) and Na_2_-EDTA, was patented by the corresponding author in 2018 (USPTO 9,925,206) and has great potential as a novel antimicrobial with broad spectrum activity against virtually all pathogenic bacteria. Regarding human use, the NaNO_2_ and/or EDTA component(s) of AB569 have separately been proven safe in studies related to the treatment of cyanide poisoning (Bebarta et al., 2017), burn wounds (Wang et al., 2015), CF lung infection (Brown et al., 1985), urinary tract infection (Birkhauser et al., 2011), chelation therapy (Lanigan and Yamarik, 2002), and cosmetics (Juzeniene et al., 2007). Furthermore, both components of AB569 have been reported to increase the efficacy of certain antibiotics that are commonly used to treat a variety of infections (Zemke et al., 2014; Lebeaux et al., 2015; McDaniel et al., 2016), and individually to enhance the process of wound healing (Weller and Finnen, 2006; Wang et al., 2015). Our recent work in Proc. Natl. Acad. Sci. (McDaniel et al., 2020) showed that AB569 has excellent bactericidal activity against all tested Gram-positive (G^+^) and Gram-negative (G^–^) bacteria including those that are MDR. Importantly, in that study, we also observed no discernable toxicity of AB569 to human airway (e.g., CF), skin (e.g., burn), or bladder (e.g., UTI’s) cells or in a mouse model of PA airway infection and no development of resistance by bacteria cultured *in vitro* (McDaniel et al., 2016, 2020).

## Conclusion

The global pandemic of antibiotic resistance has been an ever-increasing problem for decades and has been nearly “forgotten” due to the COVID-19 pandemic which has taken scientific and political priority for nearly ∼1.5 years (statement as of 3-27-21 based upon the “discovery” of SARS-CoV-2 in 2019). Drug companies have seemingly always been “behind the eight ball” in the development of novel antimicrobials that are not only bactericidal or bacteriostatic, but also offer the organisms little hope for development of resistance to them. The organisms inevitably are always a few “steps ahead” of the substantial creative brain power of the world’s best scientists. Thus, there is only a paltry glimmer of hope for control and, above all, eradication of such life-threatening organisms. We strongly believe that AB569 holds such promise.

AB569 is potentially one solution to the above problem. Although many organisms can metabolize nitrite, they are generally sensitive to it even under slight acidic conditions (e.g., pH 6.5). Organisms that cannot or have difficulty metabolizing nitrite are even more susceptible (Yoon et al., 2006). In contrast, there are only ∼4–8 organisms that have been shown to degrade EDTA including *Chelativorans multitrophicus* (Doronina et al., 2010), *C. oligotrophicus* (Doronina et al., 2010), *Aminobacter aminovorans* (Yuan and VanBriesen, 2008), and *Mesorhizobium*, none of which are considered human pathogens and are only capable of aerobic respiration (denitrification does not occur) (Willems, 2014).

**FIGURE 6:**
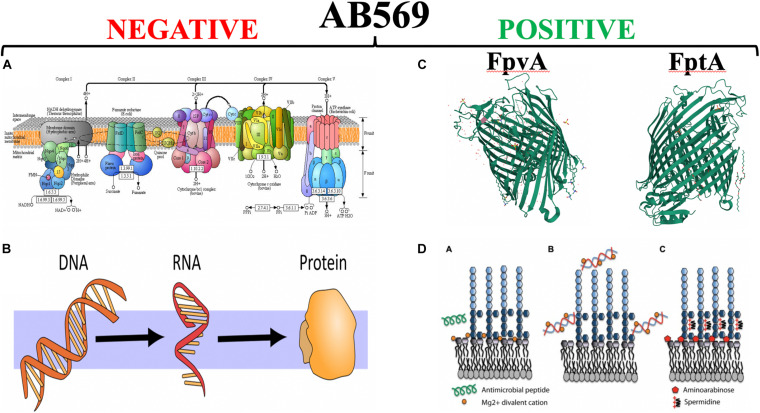
The left panel side **(A,B)** represent those vital processes that are significantly down-regulated by AB569. **(A)** The bacterial; respiratory chain reprinted from Kanehisa Laboratories and the KEGG project: www.kegg.org. **(B)** The processes of DNA, RNA and protein synthesis (from https://scasource.net/2019/09/06/snapshot-what-is-rna). The right panel **(C,D)** represent those vital processes that are significantly up-regulated by AB569. **(A)** The bacterial; respiratory chain reprinted from **(C)** The X-ray crystallographic structures of the ferripyoverdine receptor FpvA (Cobessi et al., 2005a, https://www.uniprot.org/uniprot/P48632#structure) and the ferripyochelin receptor FptA (www.rcsb.org/search?request=%7B%22query%22%3A%7B%22 parameters%22%3A%7B%22attribute%22%3A%22struct.title%22%2C%22operator%22%3 A%22contains_phrase%22%2C%22value%22%3A%22Pyochelin %20outer%20membrane%20receptor%20FptA%20from%20Pseudomonas%20aeruginosa%22%7D%2C%22type%22%3A%22terminal%22%2C%22service %22%3A%22text%22%2C%22node_id%22%3A0%7D%2C%22return_type%22%3A%22entry%22%2C%22request_options%22%3A%7B%22pager%22%3A% 7B%22start%22%3A0%2C%22rows%22%3A100%7D%2C%22scoring_strategy%22%3A%22combined%22%2C%22sort%22%3A%5B%7B%22sort_by%22% 3A%22score%22%2C%22direction%22%3A%22desc%22%7D%5D%7D%2C%22request_info%22%3A%7B%22src%22%3A%22ui%22%2C%22query_id%22% 3A%22a4fddd89af57acfc2bb3e4b7b4070ee6%22%7D%7D). **(D)** Lipopolysaccharide (LPS) modifications in the presence of extracellular DNA that contribute to antimicrobial peptide resistance. **(A)** Divalent metal cations including Mg^2+^ (orange) bind to the negatively charged phosphates of the lipid A moiety of LPS and act to stabilize LPS. Antimicrobial peptides (green) can displace cations and disrupt membrane integrity, leading to cell lysis and death. **(B)** Extracellular DNA binds and sequesters cations from the environment and the membrane. **(C)** In response to limiting Mg^2+^ or cation chelation, the PhoPQ/PmrAB systems are activated leading to the production of covalently attached aminoarabinose to the phosphates of lipid A (red) and the production of polycation spermidine (charge, + 3) on the surface, which may bind electrostatically to negative charges in the core oligosaccharide (dark blue) of the O antigen. Both modifications mask the negative charges and protect the outer membrane from peptide damage (Lewenza, 2013) https://www.researchgate.net/figure/Lipopolysaccharide-LPS-modifications-in-the-presence-of-extracellular-DNA-that_fig1_235658570, with the red “X” through it is to indicate that AB569 dramatically downregulates genes involved in DNA, RNA, protein and ATP biosynthesis. In contrast, the right panel shows the ribbon diagrams of the PA ferripyochelin (FptA, top) and ferripyoverdin (FpvA, bottom, receptors).

Finally, the level of mechanistic complexity of AB569 bactericidal activity is multifactorial. Upon exposure of anaerobically grown *PA* (reminiscent of late-stage chronic CF airway infections), RNA-seq analyses clearly indicate a dramatic and paralyzing decrease in transcription of essential genes involved in DNA (e.g., ribonucleotide reductase), RNA, protein (e.g., ribosomal proteins), electron transport chain enzymes (e.g., cytochrome c oxidase, succinate dehydrogenase) and ATP synthesis (Figure 6, red minus sign). In contrast, there was a marked up-regulation of genes involved in iron regulation, and siderophore synthesis/acquisition machinery (McDaniel et al., 2020). We are currently trying to secure support to move the AB569 technology forward toward animal toxicology, phamacodyanamic/pharmacokinetic data and ultimately human clinical trials.

## Author Contributions

DH wrote the initial and final draft. LS provided various segments on burns and wounds. PS, RK, NK, and HK provided expertise in quorum sensing, and structural biology and translational medicine. All authors contributed to the article and approved the submitted version.

## Conflict of Interest

The authors declare that the research was conducted in the absence of any commercial or financial relationships that could be construed as a potential conflict of interest.

## Footnotes

1 www.defense.gov/casualty.pdf

2 https://www.cdc.gov/drugresistance/us-activities.html
